# Circular RNA MAN2B2 promotes cell proliferation of hepatocellular carcinoma cells via the miRNA-217/MAPK1 axis

**DOI:** 10.7150/jca.36500

**Published:** 2020-03-05

**Authors:** Xiaoying Fu, Juanjuan Zhang, Xing He, Xu Yan, Jian Wei, Min Huang, Yaya Liu, Jianwei Lin, Hongxing Hu, Lei liu

**Affiliations:** 1 Shenzhen Third People's Hospital, Second Affiliated Hospital of Southern University of Science and Technology.; 2 Shenzhen University.; 3 Shenzhen People's Hospital. Shenzhen 518039, China

**Keywords:** hepatocellular carcinoma, circMAN2B2, miR-217, MAPK1, circular RNA

## Abstract

The increasing incidence of hepatocellular carcinoma (HCC) is a major challenge worldwide. In the past few years, an increasing number of studies have suggested that circular RNAs (circRNAs) play an important role in the development of human tumors, including HCC, but our understanding of their function is still limited. In this study, we investigated differences in the expression of circRNA MAN2B2 (circMAN2B2) in hepatocellular tissues and paired normal tissues. We found that knockdown of circMAN2B2 expression in the HCC cell lines Hep-G2 and Huh-7 significantly inhibited cell proliferation by sponging (miRNA) miR-217 and inhibiting its function. Through a series of experiments, we also demonstrated that miR-217 functioned as a tumor suppressor molecule in HCC cells and regulated the expression of mitogen-activated protein kinase 1 (MAPK1). Restoration of MAPK1 rescued repression of cell proliferation induced by circMAN2B2 knockdown. In summary, our study indicated that circMAN2B2 acted as an onco-miRNA in HCC by sponging miRNA-217 to promote MAPK1 expression.

## Introduction

Hepatocellular carcinoma (HCC) is the most common primary liver tumor and is one of the most challenging cancers among human malignant tumors 1. The mortality rate of HCC is high and its incidence is steadily increasing. Despite advances in the early diagnosis and treatment of HCC, long-term survival remains poor due primarily to a prohibitively high rate of recurrence 2. Therefore, there is an urgent need to elucidate the molecular regulatory mechanisms of HCC to improve the diagnosis, treatment, and overall prognosis of the disease.

Circular RNAs (circRNAs) are non-coding RNAs with a covalent loop structure that perform biological functions through a variety of regulatory modes. For example, circRNAs can affect the transcription and translation of coding genes by modulating the transcription of messenger RNAs (mRNAs) and alternative splicing of precursor mRNAs 3. In addition, circRNA can also act as an endogenous competitive molecular sponge for microRNAs (miRNAs) or other types of RNA to regulate the flow of RNA-related information in cells 4. Previous studies have shown that circRNAs play a regulatory role in the malignant phenotype of HCC. For example, circADAMTS13 serves as an endogenous sponge of miR-484, and its abnormally low expression in the HCC resulted in abnormal proliferation of cancer cells 5. The exosomal circRNA secreted by adipocytes promotes the growth of HCC by inhibiting miR-34a and activating the USP7 / Cyclin A2 signaling pathway 6.

The dysregulation and function of miRNAs have received widespread attention in studies of tumorigenesis but the relationship between miRNAs and circRNAs requires further investigation. Previous studies have demonstrated that RNA molecules have a mutually regulated relationship, as shown for example by competitive endogenous RNAs (ceRNAs) that competitively share miRNAs 7. Transcripts such as mRNAs and non-coding RNAs can serve as natural miRNA sponges by competitively binding to target miRNA response elements (MREs), which can produce an inhibitory effect on expression and function of miRNAs 8. Similar to long non-coding RNAs (lncRNAs), circRNAs contain multiple MREs which allow circRNAs to act as molecular sponges for miRNAs and regulate gene expression at the transcriptional or post-transcriptional level 9, 10. While the study of circRNAs is still in its infancy, it is now known that the expression of circRNAs is strictly controlled by the local micro-environment.

Non-coding RNAs have been reported to regulate the initiation and progression of HCC. MiRNAs and lncRNAs are two major types of non-coding RNAs that have been extensively studied for their critical role in tumor pathogenesis 11-14. In contrast, circRNAs are a new type of non-coding RNA. Although their existence has been known for some time, their functions have not been fully elucidated. CircRNAs are covalently connected and are constructed in a head-to-tail closed loop which renders them resistant to degradation in the intracellular environment 15. The structural characteristics of circRNAs make them stable in HCC tissues and good markers for diagnosis and prognosis of HCC. Circular RNA MAN2B2 (circRNA-MAN2B2 ID: has_circ_0069085) is derived from the product of MAM2B2 splicing, but its function has not been confirmed.

In this study, we analyzed the expression profile of circRNAs in HCC tissues and found that circMAN2B2 was highly expressed in HCC tissues, which was closely related to the prognosis of HCC patients. In addition, we have demonstrated through a series of experiments that circMAN2B2 can serve as an endogenous molecular sponge of miR-217, which can up-regulate the expression of the MAPK1 signaling pathway to promote cell proliferation of HCC. Inhibition of circMAN2B2 expression was confirmed in our work to significantly inhibit the development of HCC *in vitro*. As an effective cancer-associated molecular marker, circMAN2B2 has a potential role to play in the diagnosis and treatment of HCC.

## Results

### Expression of circMAN2B2 in HCC tissues and cells

**Figure 1:**
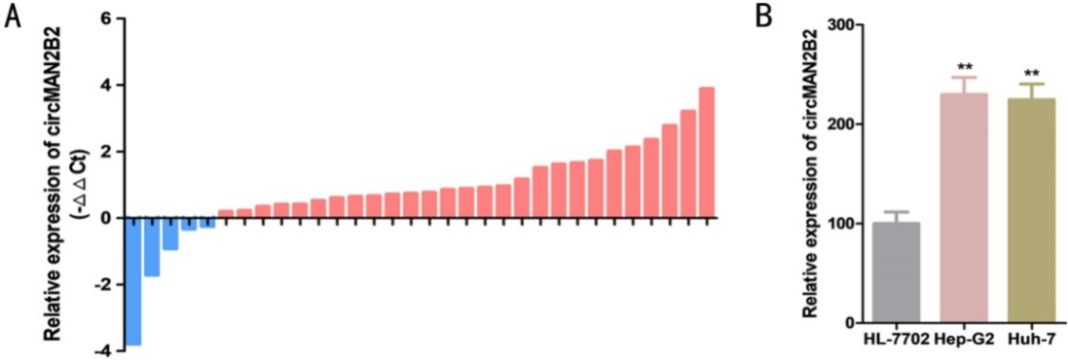
** (A)** Expression levels of circMAN2B2 in 32 HCC tissues. **(B)** Expression levels of circMAN2B2 in HCC cell lines.

The relative expression levels of circMAN2B2 were determined by real-time quantitative PCR assay (RT-qPCR) in 32 patients with HCC. Relative to paired matched normal cancer tissue, we found that the expression of circMAN2B2 was significantly greater in 84.38% of HCC tissues (27 out of 32) (Figure 1A). In cell lines, we found that circMAN2B2 was highly expressed in Hep-G2 and Huh-7 cells compared to the normal HL-7702 hepatocyte cell line. As shown in Table 1, the up regulating of circMAN2B2 was positively correlated with the TNM stage of HCC. However, gender, age, tumor size, and lymph node metastasis were not associated with circMAN2B2 expression levels. These results suggested that circMAN2B2 may play a carcinogenic role in HCC.

### Knockdown of circMAN2B2 inhibits HCC cell proliferation and invasion

**Figure 2:**
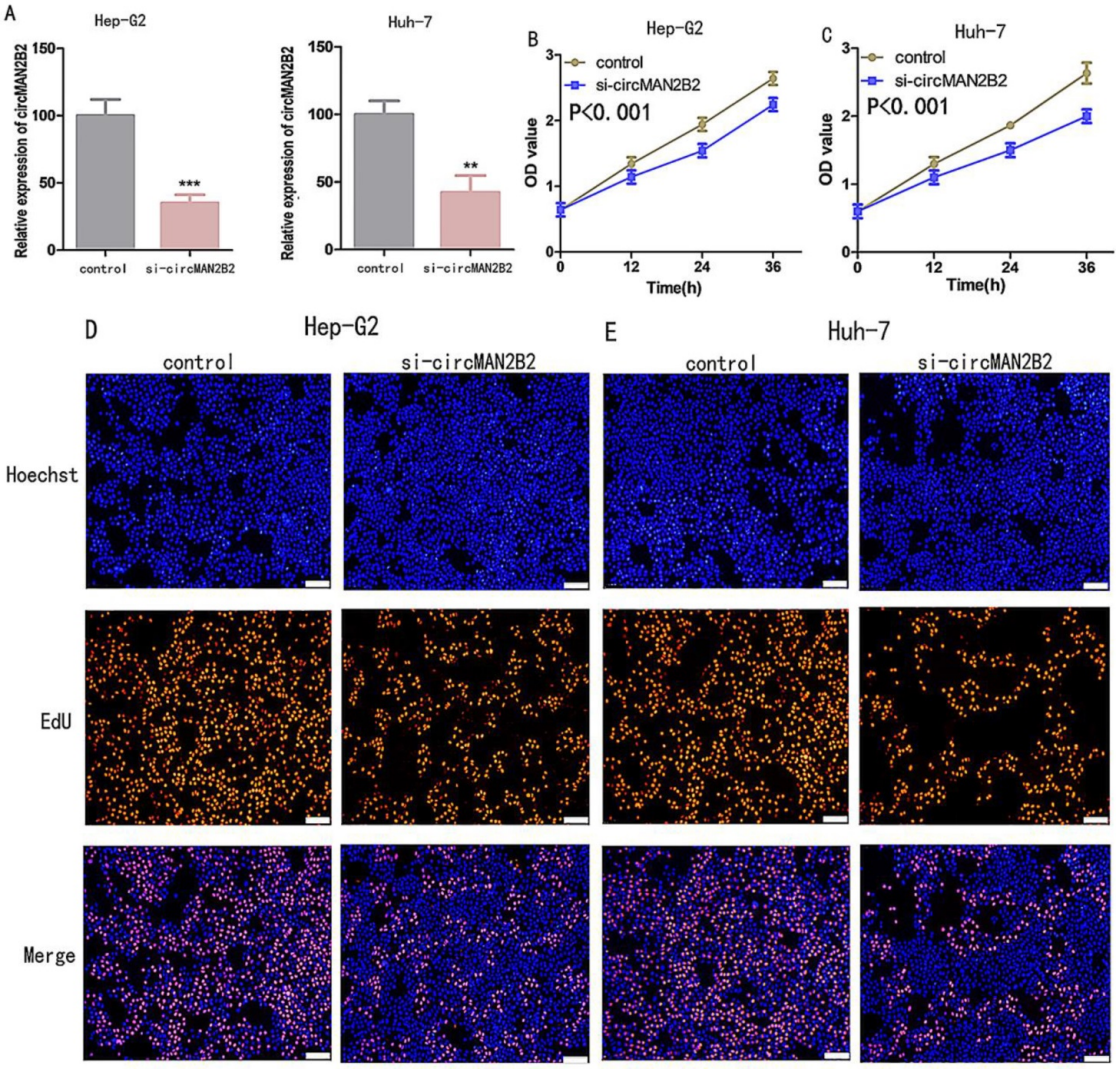

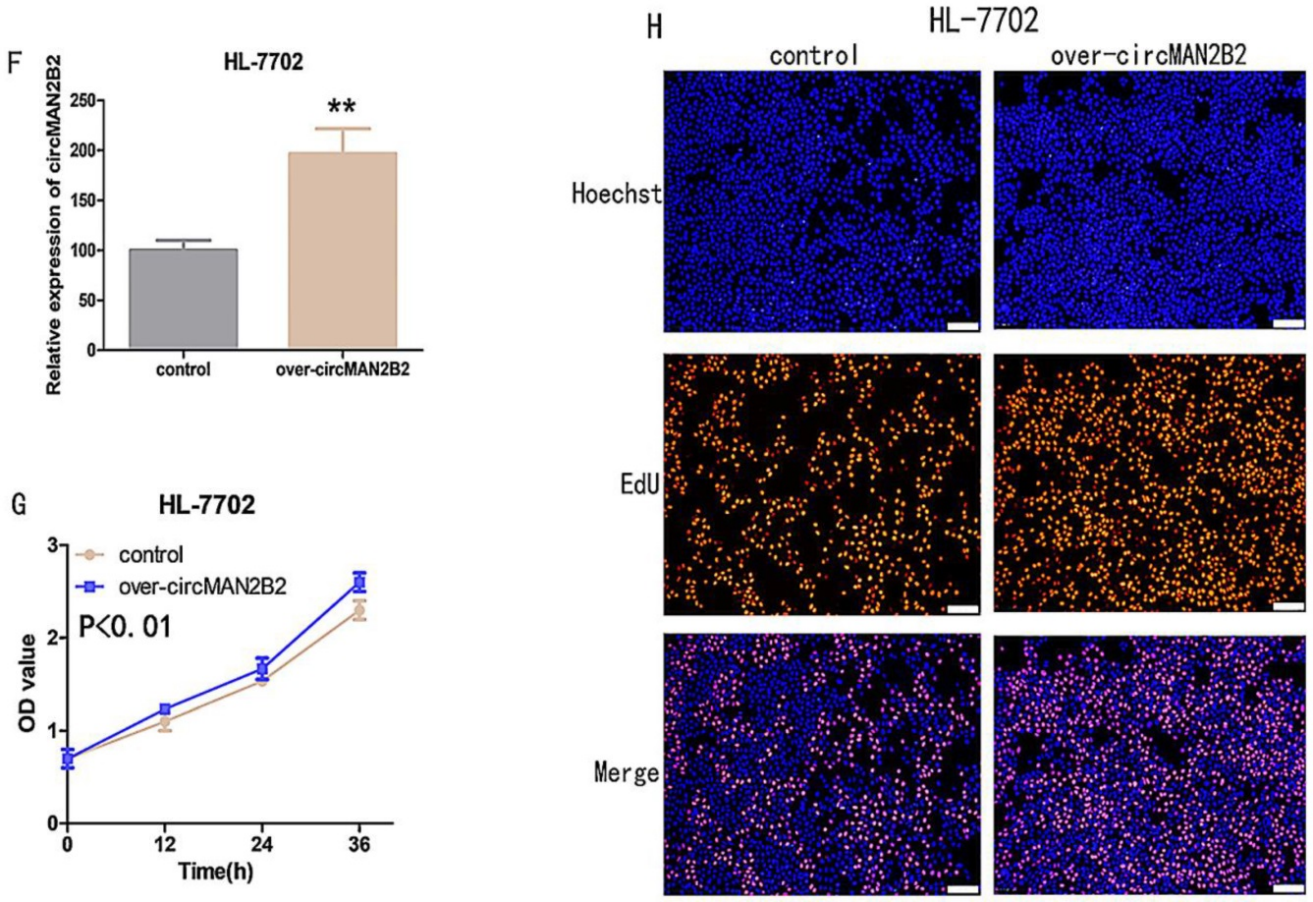
** (A)**Knockdown effects of si-circMAN2B2 in Hep-G2 and Huh-7 cells. **(B)** Cell proliferation was suppressed in Hep-G2 cells transfected with si-circMAN2B2. **(C)** Cell proliferation was suppressed in Huh-7 cells transfected with si-circMAN2B2. **(D)** Suppression of cell proliferation was observed in Hep-G2 cells transfected with si-circMAN2B2 in EdU assays. **(E)** Suppression of cell proliferation was observed in Huh-7 cells transfected with si-circMAN2B2 in EdU assays. **(F)** The effects of circMAN2B2 overexpression after transfection with over-circMAN2B2 in HL-7702 cells. **(G)** Increased proliferation of HL-7702 cells transfected with over-circMAN2B2. **(H)** Cell proliferation was increased in HL-7702 cells transfected with over-circMAN2B2. All experiments were repeated three times. Data are shown as means ± SD (*p<0.05, **p< 0.01, ***p< 0.001).

To investigate the function of circMAN2B2 in HCC, we knocked down circMAN2B2 in Hep-G2 and Huh-7 cells by transfection with specific siRNA against circMAN2B2 (si-circMAN2B2). The results of qRT-PCR showed that the expression of circMAN2B2 was significantly decreased in Hep-G2 and Huh-7 cells after siRNA transfection (Figure 2A). We then used Cell Counting Kit-8 (CCK8) and 5-ethynyl-20-deoxyuridine (EdU) assays to analyze whether down-regulation of circ-MAN2B2 affected HCC cell proliferation. CCK8 assays showed that down regulation of circMAN2B2 significantly inhibited cell proliferation of Hep-G2 (Figure 2B) and Huh-7 cells (Figure 2C). In addition, the EdU results also showed that down-regulation of circMAN2B2 significantly inhibited cell proliferation in HCC cell lines Hep-G2 (Figure 2D) and Huh-7 (Figure 2E). These data suggested that silencing of circMAN2B2 inhibited HCC cell proliferation.

Next, to further verify that circMANB2 played a role in promoting cell proliferation, we overexpressed circMAN2B2 in the HL-7702 cell line and tested its effect on cell proliferation. After transfection of the circMAN2B2 overexpression vector (over-circMAN2B2), the results of qPCR assays showed that the expression level of circMAN2B2 in HL-7702 cells was significantly increased (Figure 2F). We then used CCK-8 and EdU assays to analyze the effects of circMAN2B2 overexpression on HL-7702 cells and found that overexpression significantly enhanced HL-7702 cell proliferation (Figure 2G, Figure 2H).

### CircMAN2B2 sponges and inhibits miR-217 expression in HCC

**Figure 3:**
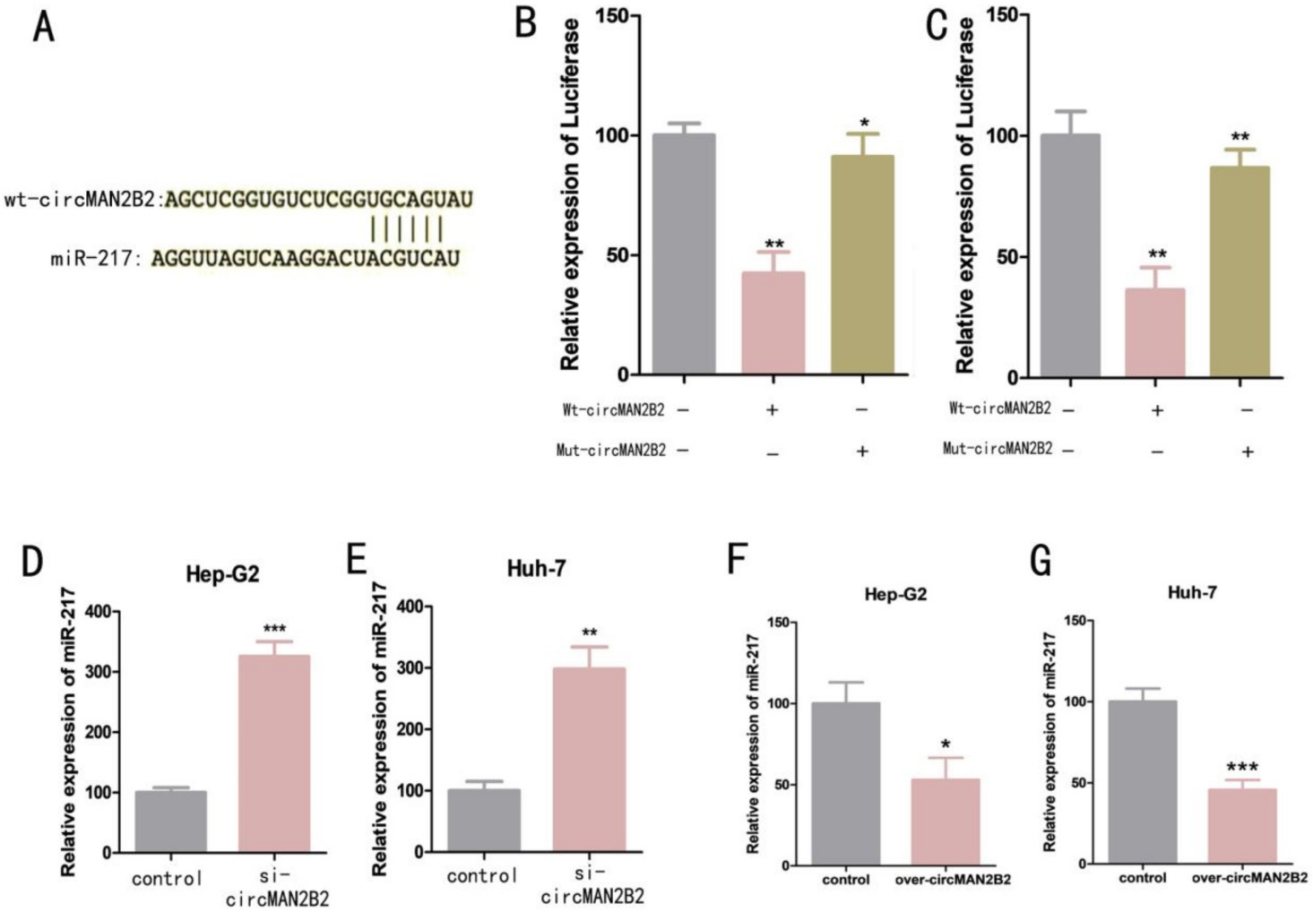
** (A)** Luciferase reporter assay analysis of the binding between miR-217 and predicted binding sites in circMAN2B2. **(B)** Effects of wt-circMAN2B2 and mut-circMAN2B2 transfection on the miR-217-associated luciferase expression vector in Hep-G2 cells. **(C)** Effects of wt-circMAN2B2 and mut-circMAN2B2 transfection on the miR-217-associated luciferase expression vector in Huh-7 cells. **(D)** The effect of inhibiting circMAN2B2 expression on the expression of miR-217 in Hep-G2 cells. **(E)** The effect of inhibiting circMAN2B2 expression on the expression of miR-217 in Huh-7 cells. **(F)** The effect of increasing circMAN2B2 expression on the expression of miR-217 in Hep-G2 cells. **(G)** The effect of increasing circMAN2B2 expression on the expression of miR-217 in Huh-7 cells. All experiments were repeated three times. Data are shown as means ± SD (*p<0.05, **p< 0.01, ***p< 0.001).

We next investigated the molecular mechanism of circMAN2B2 activity within cells. Previous studies have shown that circRNAs can serve as molecular sponges for miRNAs^16^. Bioinformatics analysis was used to predict that miR-217 was the potential target of circMAN2B2 (Figure 3A). To confirm that circMAN2B2 binds directly to miR-217, we performed a dual luciferase reporter assay. We constructed a miR-217-associated luciferase expression vector and tested its luminescence under the influence of wild-type circMAN2B2 mimics (Wt-circMAN2B2) and mutant circMAN2B2 mimics (Mut-circMAN2B2). We found that luciferase activity did not change when Mut-circMAN2B2 alone was transfected (Figure 3B, Figure 3C). However, when both Wt-circMAN2B2 and Mut-circMAN2B2 were simultaneously transfected into the cells, luciferase activity was significantly inhibited (Figure 3B, Figure 3C). In addition, we also found that expression of miR-217 was up regulated when circMAN2B2 was knocked down in the cells (Figure 3D, Figure 3E). Interestingly, we observed a negative correlation between the levels of circMAN2B2 and miR-1275, which promotes cell proliferation and invasion of lung cancer^17^, by down and up regulating the expression of circMAN2B2 in cells (Figure 3F, Figure 3G). These data suggested that miR-217 is the direct target of circMAN2B2 in HCC.

### MiR-217 exerts its role in regulating MAPK1 expression

**Figure 4:**
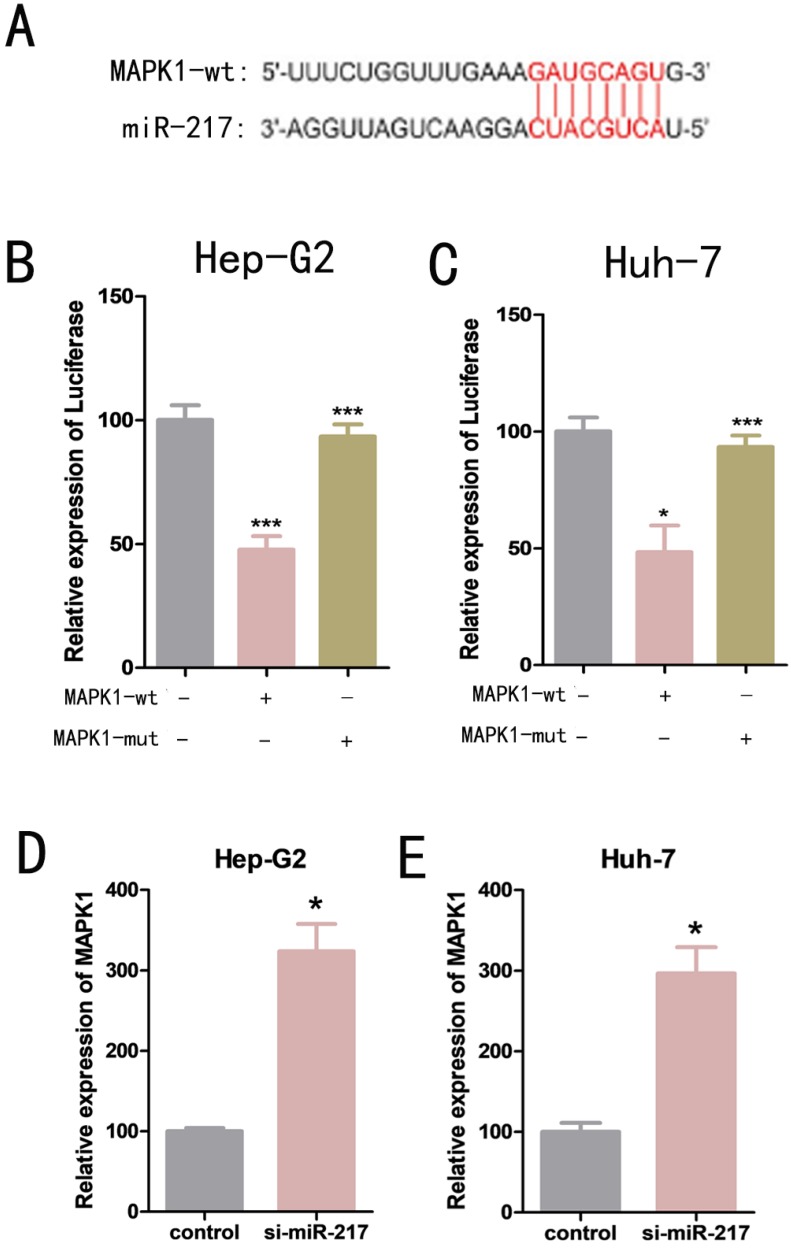
** (A)** Luciferase reporter assay analysis of the binding between MAPK1 and predicted binding sites in miR-217. **(B)** Effects of wt-MAPK1 and mut-MAPK1 transfection on the miR-217-associated luciferase expression vector in Hep-G2 cells. **(C)** Effects of wt-MAPK1 and mut-MAPK1 on miR-217-associated luciferase expression vector in Huh-7 cells. **(D)** The effects of inhibiting miR-217 expression on the expression of MAPK1 in Hep-G2. **(E)** The effects of inhibiting miR-217 expression on the expression of MAPK1 in Huh-7. All the experiments were repeated three times. Data are shown as mean ± SD (*p<0.05, **p< 0.01, ***p< 0.001).

We used bioinformatics analysis to predict that mitogen-activated protein kinase 1 (MAPK1) was one of the possible downstream target genes for miR-217 (Figure 4A). Dual luciferase reporter assays were used to determine the relationship between miR-217 and MAPK1. We constructed a wild-type MAPK1-related luciferase reporter vector (MAPK1-wt) and a mutant MAPK1-related luciferase reporter vector (MAPK1-mut). We then co-transfected miR-217 mimics with MAPK1-wt or MAPK1-mut into the cells, and measured the expression level of luciferase. We found that the miR-217 mimics significantly inhibited the expression of luciferase from the MAPK1-wt vector (Figure 4B, Figure 4C), but had no obvious effect on the expression of luciferase from the MAPK1-mut vector (Figure 4B, Figure 4C). The results of qPCR suggested a negative correlation between miR-217 and MAPK1 (Figure 4D, Figure 4E).

### The circMAN2B2/miR-217/MAPK1 regulatory axis affects HCC cell proliferation and invasion

**Figure 5:**
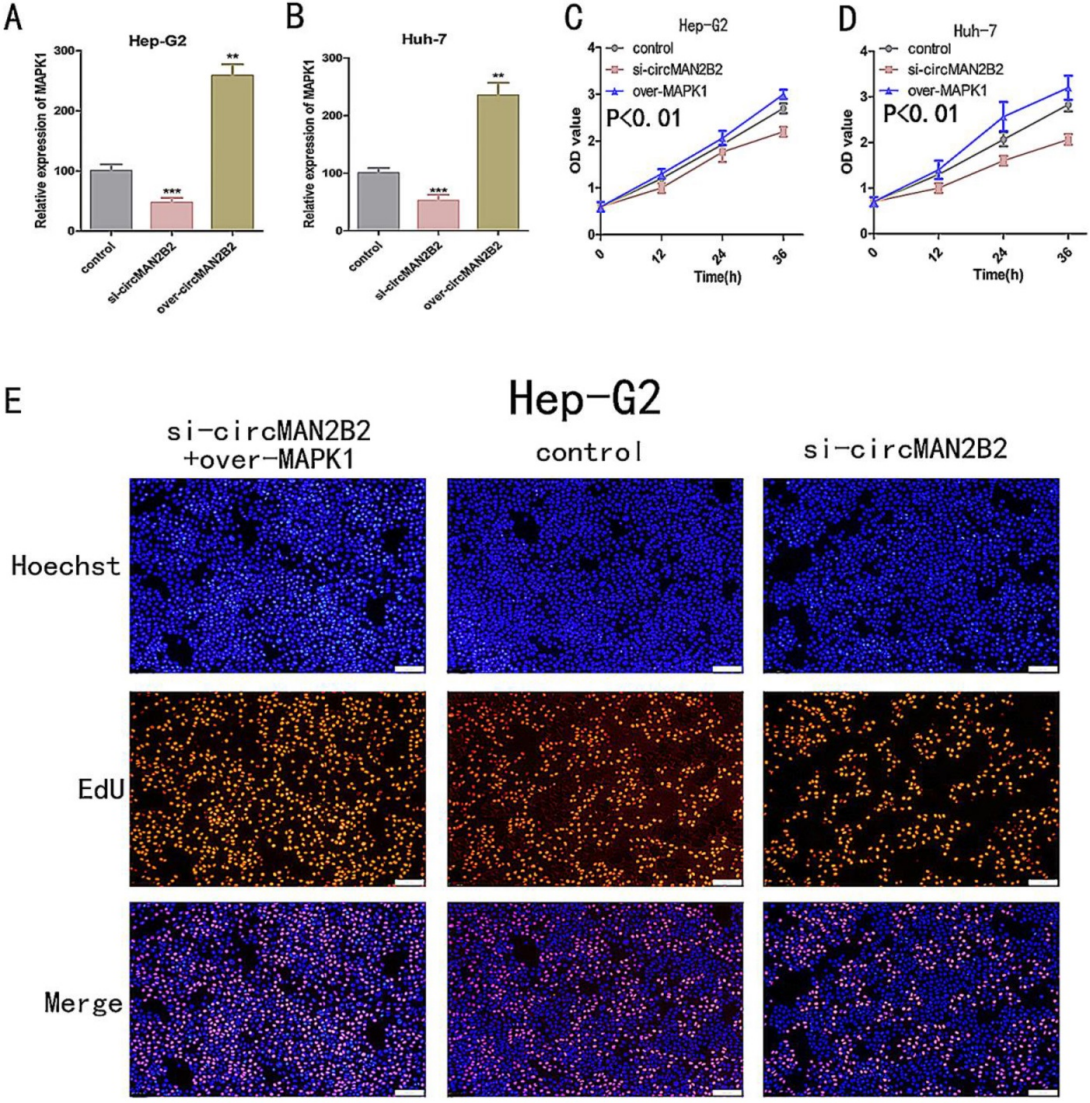

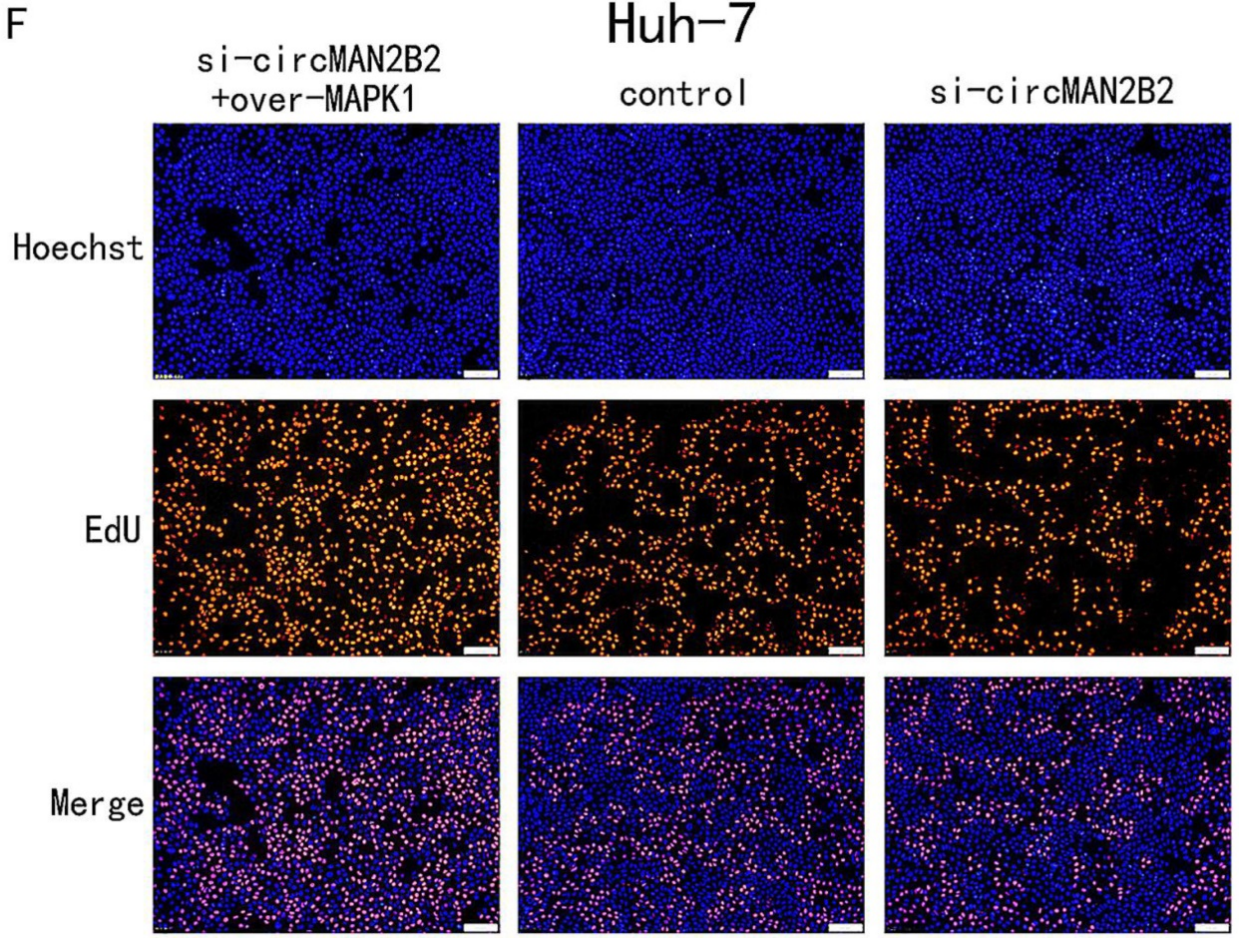
** (A)** The effect of inhibiting or increasing the expression of circMAN2B2 on the expression of MAPK1 in Hep-G2 cells. **(B)** The effect of inhibiting or increasing the expression of circMAN2B2 on the expression of MAPK1 in Huh-7 cells. **(C)** Increasing the expression of MAPK1 reversed the inhibition of cell proliferation caused by suppressing circMAN2B2 in Hep-G2 cells. **(D)** Increasing the expression of MAPK1 reversed the inhibition of cell proliferation caused by suppressing circMAN2B2 in Huh-7 cells. **(E)** Increasing the expression of MAPK1 reversed the inhibition of cell growth caused by suppressing circMAN2B2 in Hep-G2 cells. **(F)** Increasing the expression of MAPK1 reversed the inhibition of cell growth caused by suppressing circMAN2B2 in Huh-7 cells. All experiments were repeated three times. Data are shown as means ± SD (*p<0.05, **p< 0.01, ***p< 0.001).

In the above study, we showed that circMAN2B2 directly regulated miR-217 and that miR-217 inhibited MAPK1 expression in HCC cells. Therefore, we hypothesized that circMAN2B2 promoted the proliferation of HCC cells through the circMAN2B2/miR-217/MAPK1 signaling pathway. To demonstrate this, we used qPCR experiments to determine whether circMAN2B2 upregulated MAPK1 expression. We found that when expression of circMAN2B2 was inhibited by siRNA in Hep-G2 and Huh-7 cells, the expression of MAPK1 was down regulated (Figure 5A, Figure 5B). In addition, the results of CCK-8 (Figure 5C, Figure 5D) and EdU assays (Figure 5E, Figure 5F) showed that overexpression of MAPK1 reversed the inhibition of cell proliferation caused by the knockdown of circMAN2B2. Finally, we sought to investigate the regulatory role of the circMAN2B2/miR217/MAPK1 signaling pathway in HCC. According to the results of CCK-8 (Figure 5C, Figure 5D) and EdU assays (Figure 5E, Figure 5F), regulation of the circMAN2B2/ miR-217/MAPK1 signaling axis significantly affected the proliferation of Hep-G2 and Huh-7 cells. Taken together, these data demonstrated that circMAN2B2 played a role in regulating cell proliferation in HCC by regulating the expression of miR-217/MAPK1.

## Discussion

As a highly heterogeneous malignant tumor, the molecular mechanism of HCC development remains unclear. Limited treatment options and poor surgical outcomes remain an obstacle to overcoming HCC. Recently, non-coding RNAs have received much attention in cancer research. Numerous studies have demonstrated that the expression of non-coding RNAs (including lncRNAs, miRNAs and circRNAs) is abnormal in many types of cancer and is involved in epigenetic regulation during cancer development 18. Several studies have demonstrated that tumor-specific non-coding RNA can serve as markers for survival prediction and therapeutic evaluation in cancer patients 19, 20.

A growing number of studies have reported that aberrantly expressed circRNAs can serve as ceRNAs to regulate miRNAs and play a key role in the development and progression of malignant tumors 21, 22. In our study, bioinformatics analysis and luciferase reporter gene analysis demonstrated that miR-217 was a target of circMAN2B2 in HCC cells. By modulating the expression levels of circMAN2B2 and miR-217, we found that circMAN2B2 negatively regulated miR-217 expression. According to ceRNA theory, we believe that circMAN2B2 may act as a molecular sponge to negatively regulate miR-217, which plays a role in promoting proliferation of HCC cells. A previous study has shown that circMAN2B2 increases the expression of FOXK1 by interacting with miR-1275, which promotes cell proliferation and invasion of lung cancer^17^. The results of our study are consistent with these results. In addition, to further explore the molecular regulatory network of circMAN2B2/miR-217, we examined MAPK1 as the potential downstream target of miR-217. Previous studies have confirmed that up-regulation of MAPK1 can strongly promote the development of malignant tumors^23^, ^24^. In our study, we demonstrated that MAPK1 is a target of miR-217 through luciferase reporter and qPCR assays and confirmed that circMAN2B2 promotes HCC cell proliferation through the miR-217/MAPK1 regulatory axis.

This is the first study to the best of our knowledge to demonstrate the function and the molecular mechanism of circMAN2B2 in HCC. In our work, we illustrated the expression profiles and clinical feature of circMAN2B2 in HCC, and further investigated its potential downstream signal axis in HCC. CircMAN2B2 is a promising biomarker for diagnosis and treatment of HCC. We found that the expression of circMAN2B2 was significantly greater in HCC tissues and that this higher level of expression was positively correlated with HCC histological grade and TNM stage. In addition, we further investigated the role of circMAN2B2 in HCC and found that inhibiting the expression of circMAN2B2 inhibited HCC cell proliferation. We overexpressed circMAN2B2 in HL-7702cells and found that overexpression promoted cell proliferation. Therefore, targeting circMAN2B2 may provide a novel target for the treatment and diagnosis of HCC.

In summary, circMAN2B2 was identified as an onco-miRNA which played a key role in promoting HCC cell proliferation. In addition, for the first time to our knowledge, our study has elucidated the interaction between circMAN2B2, miR-217 and MAPK1, revealing a novel mechanism by which circMAN2B2 positively regulates MAPK1 expression in HCC by sponging miR-217. Our results indicated that circMAN2B2 serve as specific target affecting the cell proliferation of HCC. Maybe, it can be developed as a novel and more effective therapeutic target for HCC. This may change the current dilemma of advanced HCC treatment. In addition, with the development of artificial gene circuit 25, we can design circMAN2B2 specific gene circuits for HCC specific treatment.

## Methods

### Patient samples

We enrolled 32 HCC patients who received surgical treatment for our study. HCC tissues and paired normal tissues were snap-frozen in liquid nitrogen immediately after resection. Informed consents were obtained from all patients. Our study was approved by the institutional research ethics committee of Shenzhen Second People Hospital (Shenzhen, China).

### Cell lines and cell culture

All cell lines used in our study (HL-7702, Hep-G2 and Huh-7) were purchased from the Institute of Cell Biology, Chinese Academy of Sciences (Shanghai, China). The cells were cultured in DMEM medium (Invitrogen, Carlsbad, CA, USA) supplemented with 10% fetal bovine serum and 1% antibiotics (100µg/ mL streptomycin sulfate and 100 U/mL penicillin) in an atmosphere of 5% CO_2_ at 37°C.

### Cell transfection

Cell transfection was performed using lipofectamine 3000 Transfection Reagent (Invitrogen, Carlsbad, CA, USA). Transfections were performed following the manufacturer's protocol when the cells reached 70%-80% confluency.

### Real-time quantitative PCR

Total RNA from cells and tissues was extracted using TRIzol reagent (Invitrogen, Grand Island, NY, USA) according to the protocol of manufacturer. The PrimeScript RT Reagent Kit with gDNA Eraser (Takara, Dalian, China) was used to convert total RNA into cDNA following the protocol of manufacturer. RT-qPCR was performed using a standard SYBR Green PCR kit (Takara, Dalian, China) according to the protocol of manufacturer. PCR reactions were measured using the ABI PRISM 7500 Fluorescent Quantitative PCR System (Applied Biosystems, Foster City, CA, USA). All primers used in our study are shown in Table 2.

### Cell proliferation assays

We evaluated the cell proliferation using EdU assays (Ribobio, Guangzhou, China) and CCK-8 assays (Beyotime Institute of Biotechnology, Shanghai, China). For the EdU assay, the procedure followed the manufacturer's protocol. For CCK-8 assays, we incubated the cells in a 96 well plate for 24 h. Absorbance in each well was measured by a microplate reader (Bio-Rad, Hercules, CA, USA) at 0, 24, 48, and 72 h after transfection.

### Statistical analysis

All assays were repeated in triplicate. Data are shown as means ± standard deviation (SD). We performed all statistical analyses using SPSS 20.0 software (IBM, Armonk, NY, USA). circMAN2B2 expression differences between HCC cancer tissues and paired normal tissues were analyzed using paired samples *t*-test. CCK-8 assay data were analyzed by ANOVA. Analyses of other data were performed by independent samples *t*-tests. A P-value less than 0.05 was considered statistically significant.

## Figures and Tables

**Table 1:** Correlation between circMAN2B2 expression and clinicopathological characteristics of hepatocellular carcinoma patients

Features	Total	circMANB2 expression		P value
		Low	High	
**Gender**				
Female	10	3(30.0%)	7 (70.0%)	0.454
Male	22	2 (9.1%)	20 (90.9%)	
**Age**				
≤60	12	0 (0.0%)	12 (100.0%)	0.581
>60	20	5 (25.0%)	15 (75.0%)	
**Histological grade**				
Low	9	3 (33.3%)	6 (66.7%)	0.004
High	23	2 (8.7%)	21 (91.3%)	
**Lymph node metastasis (N)**				
N0	25	4 (16.0%)	21 (84.0%)	0.129
N1, N2, N3	7	1 (14.3%)	6 (85.7%)	
**TNM stage**				
0/I	20	5 (25.0%)	15 (75.0%)	0.151
II/III/IV	12	1 (8.3%)	11(91.7%)	

*P < 0.05 was considered significant (Chi-square test between 2 groups)

**Table 2:** The primers used in our study.

Gene	Sequences (5' → 3')
circMAN2B2	F: GCCAAGATCAATCCTCCATGAGTAGTG
	R: TCCACGGTCCTGCTGTCCATAG
miR-217	F: TTGAGGTTGCTTCAGTGA
	R: GGAGTAGATGATGGTTAGC
MAPK1	F: GGTGCCTCCTCTTGACTTCC
	R: AACCTGAACCTGACTGTCCATT
GADPH	F: AACGGATTTGGTCGTATTG
	R: GGAAGATGGTGATGGGATT
